# Preliminary Schools for Probationers

**Published:** 1909-04-17

**Authors:** 


### Nursing Administration.

### PRELIMINARY SCHOOLS FOR PROBATIONERS.

### IV.—THE CURRICULUM.

Very few matrons who are accustomed to the stupidities of the ordinary probationer and her slow rate of progression in the things which belong to her calling have any idea of the amount which can be taught to the very same woman under better conditions. The examinations set in many training schools at the end of one or two years' instruction in physiology might seem to indicate that the mental calibre of the probationers was of no high order. And yet it is not so much that the probationers are slow to learn. It is that their opportunities for sustained work are scanty, that they come jaded to their lectures, find little time to revise their notes, and live generally in that whirl of bodily labour which is the worst possible condition for acquiring abstract knowledge. Given proper instruction, a routine in which study is the aim instead of the interruption in the day's routine, and a calm environment, and all that is uncomfortably worried through in the space of years may be far better mastered in the space of a few weeks.

The theoretical subjects which the preliminary pupil is set to study are as a rule elementary anatomy, physiology, and hygiene. There can be no question that these are subjects better begun under the guidance of a competent sister than attacked in the lecture room. Indeed, most matrons find that it is necessary to supplement lectures from the medical staff by very easy explanations in class. The study of science is so generally neglected in the school course that its beginnings prove very arduous to the average probationer. Taking the preliminary course at six weeks, the seventh week being occupied by examinations, it will be seen that two lessons a week on each of the three subjects mentioned will be necessary to fit in courses of twelve lessons. Each lesson will last an hour, and be supplemented by two hours' private study, so that at least 18 hours a week will be given up to theoretical work. This may not seem a great deal. But three hours a day of consecutive study will carry the pupil a long way. It is perhaps the most important part of the preliminary training, because while practical details are readily mastered even in the stress of hospital work, the theory cannot be properly grasped except in lessons following in frequent succession, so that one helps to drive in the next.

Household work is the next point. At Guy's school the admirable plan is followed of setting the pupils to do the work of their own department. The maid attached to the school attends to the floors, windows, and grate cleaning. All else is done under supervision by the pupils, who learn in this way to clean "brights," make beds, light fires, sweep, dust, etc.

The system not only has the very practical advantage of saving labour, but also makes the instruction far more interesting. It is the defect of all housekeeping and housework schools that the pupils have to have work made for them, a process which takes all the zest out of well performed duties.

The third branch of work to which pupils are introduced has some relation to the work of the wards. They are instructed in bandaging, the use of instruments, the rudiments of dispensing, and the preparation of dressings.

Lastly, the student goes through a course of systematic instruction in cookery, not merely attending lectures or demonstrations, but working in the kitchen and preparing and serving meals. At Guy's the excellent plan is adopted of setting the pupils to prepare meals for the nurses' sick-room. With so large a staff there are generally one or two invalids, and the delicacies prepared under the cookery instructor are highly relished. This valuable part of the pupils' preparation for nursing is a considerable asset in her training, and is a very popular part of the course. Unless the sister in charge is highly skilled in cookery demonstrations, it is better to engage a good teacher for this work.

The following time-table of the pupils' day at Guy's gives a good idea of the practical working-out of the scheme of instruction we have briefly outlined:—

GUY'S HOSPITAL.

Preliminary Training School.

*Time Table.*

| | |
|---|---|
| 7 A.M. | Called. |
| 7.30 A.M. | Breakfast. |
| 8 A.M. | On duty. Practical work, bedmaking, sweeping, dusting, cleaning, etc. |
| 9.30 A.M. | Chapel. |
| 9.45 A.M. | Finish practical work. |
| 10.30 A.M. | Class. Charts, poultices, etc., etc., etc. |
| 11.30 A.M. | Stock work, making sponges, pads, padding splints, etc., etc. |
| 12.15 P.M. | Dinner. |
| 1 P.M. | Study. |
| 2 P.M. | Off duty, 1 hour or 3 hours; alternate days if on duty, stock work. |
| 5 P.M. | Tea. |
| 5.30 P.M. | Class. Physiology, anatomy, or hygiene. |
| 7 to 9 P.M. | Study. |
| 9 P.M. | Supper. |
| 9.30 P.M. | Chapel. |
| 10 P.M. | In rooms. Lights out 10.45 P.M. |

On Saturday afternoons, class from 3 to 4 P.M.

Cooking classes, Tuesday and Wednesday afternoons 2 to 4 P.M.

Nurses' sick-room meals are prepared and served by the pupils.

Every alternate Sunday, off duty from 10 A.M. to 9.30 P.M.

In all essentials the various systems in vogue at different hospitals for the preparation of the probationer agree.

It will be seen that except for the third branch of the curriculum—the lessons in bandaging, use of instruments, etc.—there is nothing in the instruction which could not be given outside the hospital. Yet it is very rare to find probationers who thoroughly understand house work and sick-room cookery and have mastered the elements of physiology and hygiene, and unless they are prepared before being sent into the wards they prove, with the best intentions, a stumbling-block to all the busy workers around them.

